# High contribution and impact of resistant gram negative pathogens causing surgical site infections at a multi-hospital healthcare system in Saudi Arabia, 2007–2016

**DOI:** 10.1186/s12879-020-4939-6

**Published:** 2020-04-07

**Authors:** Aiman El-Saed, Hanan H. Balkhy, Majid M. Alshamrani, Sameera Aljohani, Asim Alsaedi, Wafa Al Nasser, Ayman El Gammal, Saad A. Almohrij, Ziyad Alyousef, Sara Almunif, Mohammad Alzahrani

**Affiliations:** 1 Infection Prevention and Control Department, King Abdulaziz Medical City (KAMC), Ministry of National Guard Health Affairs (MNGHA), P.O. Box 22490, 11426 Riaydh, Kingdom of Saudi Arabia; 2 King Saud bin Abdulaziz University for Health Sciences, Riyadh, Saudi Arabia; 3 Community Medicine Department, Faculty of Medicine, Mansoura University, Mansoura, Egypt; 4 King Abdullah International Medical Research Center, Riaydh, Saudi Arabia; 5 Department of Pathology and Laboratory Medicine, KAMC, Riyadh, Saudi Arabia; 6 Infection Prevention and Control Department, KAMC, MNGHA, Jeddah, Saudi Arabia; 7 Infection Prevention and Control Department, Imam Abdulrahman bin Faisal Hospital, MNGHA, Dammam, Saudi Arabia; 8 Infection Prevention and Control Department, King Abdulaziz Hospital, MNGHA, Al hassa, Saudi Arabia; 9 Department of Surgery, KAMC, MNGHA, Riyadh, Saudi Arabia; 10 Department of Surgery, KAMC, MNGHA, Jeddah, Saudi Arabia

**Keywords:** Antimicrobial resistance, Multidrug resistance, Surgical site infections, Surveillance, Pathogens, Hospital, Saudi Arabia

## Abstract

**Background:**

Despite being largely preventable, surgical site infections (SSIs) are still one of the most frequent healthcare-associated infections. The presence of resistant pathogens can further augment their clinical and economic impacts. The objective was to estimate the distribution and resistance in SSI pathogens in Saudi Arabia and to compare them to the US National Healthcare Safety Network (NHSN) hospitals.

**Methods:**

Targeted SSI surveillance was prospectively conducted on several surgical procedures done between 2007 and 2016 in four hospitals of Ministry of National Guard Health Affairs. Definitions and methodology of SSI and bacterial resistance were based on NHSN.

**Results:**

A total 492 pathogens causing 403 SSI events were included. The most frequent pathogens were *Staphylococcus aureus* (22.8%), *Pseudomonas aeruginosa* (20.1%), *Klebsiella* spp. (12.2%), and *Escherichia coli* (12.2%), with marked variability between surgeries. Approximately 30.3% of *Staphylococcus aureus* was methicillin-resistant (MRSA), 13.0% of *Enterococcus* spp. was vancomycin-resistant (VRE), and 5.5% of *Enterobacteriaceae* were carbapenem resistant (CRE). The highest multidrug-resistant (MDR) GNPs were *Acinetobacter* spp. (58.3%), *Klebsiella* spp. (20.4%) and *Escherichia coli* (16.3%). MRSA was significantly less frequent while cephalosporin-resistant *Klebsiella* spp., MDR *Klebsiella* spp., and MDR *Escherichia coli* were significantly more frequent in our hospitals compared with NHSN hospitals.

**Conclusion:**

GNPs in a tertiary care setting in Saudi Arabia are responsible for more than 60% of SSI with more resistant patterns than Western countries. This information may be critical to secure resources and ensure support for caregivers and healthcare leaders in implementing antimicrobial stewardship programs and evidence-based SSI preventive practices.

## Background

Surgical site infection (SSI) is a global healthcare problem increasing patient morbidity, mortality, and healthcare cost [1, 2]. Despite the fact that more than 50% are preventable [3], SSI remains the most frequent healthcare-associated infections (HAIs) in low and middle income countries, affecting up to 30% of the patients undergoing surgery [1, 4]. Even in high income countries, it is still the second frequent type of HAI, accounting for more than 20% of all HAIs [5]. Several surgical-related practices have been linked to the development of antimicrobial resistance [6]. The presence of resistant pathogens has been shown to augment the clinical and economic impacts of SSI [7]. Therefore, recent SSI guidelines stressed on the appropriate use of antimicrobial prophylaxis to reduce the risk of antimicrobial resistance [6].

Gram positive pathogens (GPPs) are traditionally the most frequently isolated SSI pathogens, with a considerable number of these pathogens are now resistant [5, 8, 9]. For example, 44% of *Staphylococcus aureus* and 20% of *Enterococcus* spp. isolated from surgical wounds in the USA are resistant [8, 9]. Unlike the prevalence of SSI in Saudi Arabia, very few studies focused on the causative pathogens and/or their resistance patterns [10–12]. Moreover, data on surgery-specific pathogens in these reports were absolutely lacking or retrieved from a small number of patients [10–12]. Likewise, very limited data on the resistance patterns of pathogens causing SSI were reported in the Gulf Cooperation Council (GCC) [10] and the Middle Eastern countries [13, 14]. For example, a study that combined aggregate SSI data from 30 developing countries (including some regional ones) could not present data on pathogen profile nor bacterial resistance [15]. The objective of the current study was to estimate the prevalence and extent of resistance of SSI pathogens identified during HAI surveillance in four tertiary care hospitals in Saudi Arabia; additionally, to compare such data to the US National Healthcare Safety Network (NHSN) hospitals.

## Methods

### Setting

Data were collected from four Ministry of National Guard Health Affairs (MNGHA) hospitals; King Abdulaziz Medical City-Riyadh (KAMC-R), King Abdulaziz Medical City-Jeddah (KAMC-J), King Abdulaziz Hospital-Alhassa (KAH), Imam Abdulrahman Bin Faisal Hospital-Dammam (IABFH). MNGHA hospitals are governmentally funded tertiary care hospitals that provide free services for more than 1.5 million Saudi National Guard soldiers, employees and their families. The total bed capacity of the four hospitals is approximately 2200 beds with an average occupancy rate of 72%. Approximately 30,000 surgical procedures are conducted in MNGHA hospitals every year.

### Design

SSI surveillance was prospectively conducted on several surgical procedures performed in four MNGHA hospitals between 2007 and 2016. The surveillance was performed by trained infection preventionists using unified SSI data collection methods, similar to those of the NHSN [16]. The surveillance was targeting selected surgical procedures based on annual risk assessments, approved by the hospital infection control committee. Post-discharge surveillance data were obtained from admission and readmission records as well as surgical follow-up, outpatient clinics, and emergency visits.

### Event eligibility

All surgeries performed on adult patients and was part of the targeted surveillance plan during the study period were initially included. Same-day and outpatient surgeries were excluded. Similarly, SSI events diagnosed clinically without laboratory confirmation were then excluded.

### Infection and resistance definitions

The surveillance definitions and data collection methods followed a locally generated GCC surveillance manual [17] that was based on the NHSN definitions [16]. While rare, more than one pathogen was allowed for a single SSI event. Multidrug resistance (MDR) definitions were retrospectively calculated as per the current NHSN definitions [18] and recent NHSN reports [8, 9]. Cephalosporin-resistant *Klebsiella* spp. was defined as *Klebsiella* spp. testing non-susceptible (resistant or intermediate) to at least one cephalosporin agent (ceftazidime, cefotaxime, ceftriaxone or cefepime) [18]. Carbapenem-resistant *Enterobacteriaceae* (CRE) was defined as *Klebsiella* spp., *Escherichia coli*, or *Enterobacter* spp. testing resistant to imipenem [18]. MDR Gram negative pathogens (GNPs) were defined as pathogens testing non-susceptible (resistant or intermediate) to at least one agent in at least 3 out of 5 antimicrobial classes; aminoglycosides (amikacin or gentamicin), cephalosporins (ceftazidime, cefotaxime, ceftriaxone, or cefepime), fluoroquinolones (ciprofloxacin or levofloxacin), carbapenems (imipenem or meropenem), β-lactamase inhibitor (piperacillin or piperacillin/tazobactam) [8, 9]. Only in MDR *Pseudomonas aeruginosa*, 2 cephalosporins (cefepime and ceftazidime) rather than 4 cephalosporins (above) were considered.

### Statistical methods

Categorical variables were presented as frequencies and percentages while continuous variables were presented as means and standard deviations. Age and gender were calculated for non-duplicate patients only. The distribution of SSI pathogens and their resistance patterns were presented by (name and wound class of) surgical procedures and significant differences were evaluated using chi-square test or Fisher exact test (as appropriate). The distribution of SSI pathogens and their resistance in MNGHA hospitals were compared to corresponding rates in NHSN hospitals after pooling data from two published NHSN reports [8, 9]. SPSS (Version 25.0. Armonk, NY: IBM Corp) was used for all statistical analyses.

## Results

### SSI events and patients

Out of 602 SSI events detected, 199 (33.1%) SSI events were excluded due to lack of microbiological data. As shown in Table 1, 492 pathogens causing 403 SSI events were included in the current analysis. Demographics and clinical characteristics of the included SSIs are shown in Table 1. The average age was 49.5 ± 18.0 years and approximately 70.0% of the patients were females. More than a third (37.0%) of procedures had non-clean wounds, mainly clean contaminated. The majority (75.5%) of the events were superficial incisional SSI, with 19.4% deep SSI and 5.1% organ/space SSI. Only 30.5% of SSI events were diagnosed before discharge. Approximately 6.3% of the patients with SSI events died during the same hospitalization.

**Table 1:** Demographics and clinical characteristics of surgical site infections (SSIs) in 4 MNGHA hospitals in Saudi Arabia (2007–2016)

	CSEC (*N* = 141)	COLO (*N* = 22)	CBGB (*N* = 153)	CHOL (*N* = 19)	HER (*N* = 16)	KPRO (*N* = 19)	Others (*N* = 33)	Total (*N* = 403)
**Eligibility**								
Procedures surveyed^a^	12,002 (53.9%)	181 (0.8%)	2409 (10.8%)	2696 (12.1%)	1613 (7.3%)	1312 (5.9%)	2035 (9.1%)	22,248 (100.0%)
SSI events detected^a^	232 (38.5%)	28 (4.7%)	208 (34.6%)	29 (4.8%)	31 (5.1%)	31 (5.1%)	43 (7.1%)	602 (100.0%)
SSI proportion	232 (1.9%)	28 (15.5%)	208 (8.6%)	29 (1.1%)	31 (1.9%)	31 (2.4%)	43 (2.1%)	602 (2.7%)
Included procedures	141 (60.8%)	22 (78.6%)	153 (73.6%)	19 (65.5%)	16 (51.6%)	19 (61.3%)	33 (76.7%)	403 (66.9%)
Included specimens^a^	160 (32.5%)	29 (5.9%)	194 (39.4%)	28 (5.7%)	21 (4.3%)	22 (4.5%)	38 (7.7%)	492 (100.0%)
**Hospital facility**^**a**^								
KAMC-Riyadh	9 (5.0%)	22 (12.3%)	131 (73.2%)	1 (0.6%)	3 (1.7%)	1 (0.6%)	12 (6.7%)	179 (100.0%)
KAMC-Jeddah	76 (51.4%)	0 (0.0%)	22 (14.9%)	13 (8.8%)	10 (6.8%)	13 (8.8%)	14 (9.5%)	148 (100.0%)
KAH-Al hasa	15 (75.0%)	0 (0.0%)	0 (0.0%)	0 (0.0%)	0 (0.0%)	0 (0.0%)	5 (25.0%)	20 (100.0%)
IAFH-Dammam	41 (73.2%)	0 (0.0%)	0 (0.0%)	5 (8.9%)	3 (5.4%)	5 (8.9%)	2 (3.6%)	56 (100.0%)
**Age, mean ± SD**^**b**^	31.2 ± 6.5	52.1 ± 18.0	63.9 ± 8.7	49.2 ± 15.1	51.0 ± 16.1	64.7 ± 11.4	50.9 ± 19.4	49.5 ± 18.0
**Gender**^**b**^								
Male	0 (0.0%)	12 (54.5%)	78 (52.0%)	5 (26.3%)	4 (25.0%)	8 (42.1%)	13 (39.4%)	120 (30.0%)
Female	141 (100.0%)	10 (45.5%)	72 (48.0%)	14 (73.7%)	12 (75.0%)	11 (57.9%)	20 (60.6%)	280 (70.0%)
**Admission**								
ICU	3 (2.1%)	6 (27.3%)	153 (100.0%)	1 (5.3%)	0 (0.0%)	0 (0.0%)	5 (15.2%)	168 (41.7%)
Wards	138 (97.9%)	16 (72.7%)	0 (0.0%)	18 (94.7%)	16 (100.0%)	19 (100.0%)	28 (84.8%)	235 (58.3%)
**Wound class**								
Clean	47 (33.3%)	0 (0.0%)	153 (100.0%)	0 (0.0%)	11 (68.8%)	19 (100.0%)	24 (72.7%)	254 (63.0%)
Non-clean	94 (66.7%)	22 (100.0%)	0 (0.0%)	19 (100.0%)	5 (31.3%)	0 (0.0%)	9 (27.3%)	149 (37.0%)
**SSI type**								
Superficial	115 (87.8%)	6 (27.3%)	117 (76.5%)	9 (81.8%)	8 (72.7%)	9 (56.3%)	20 (62.5%)	284 (75.5%)
Deep	14 (10.7%)	11 (50.0%)	32 (20.9%)	0 (0.0%)	3 (27.3%)	3 (18.8%)	10 (31.3%)	73 (19.4%)
Organ/space	2 (1.5%)	5 (22.7%)	4 (2.6%)	2 (18.2%)	0 (0.0%)	4 (25.0%)	2 (6.3%)	19 (5.1%)
**Diagnosis time**								
Before discharge	12 (9.7%)	13 (76.5%)	60 (40.5%)	9 (52.9%)	2 (12.5%)	3 (25.0%)	12 (40.0%)	111 (30.5%)
After discharge	81 (65.3%)	3 (17.6%)	69 (46.6%)	8 (47.1%)	10 (62.5%)	4 (33.3%)	15 (50.0%)	190 (52.2%)
On readmission	31 (25.0%)	1 (5.9%)	19 (12.8%)	0 (0.0%)	4 (25.0%)	5 (41.7%)	3 (10.0%)	63 (17.3%)
**Hospital death**								
No	101 (100.0%)	11 (61.1%)	116 (90.6%)	15 (100.0%)	13 (100.0%)	14 (100.0%)	29 (96.7%)	299 (93.7%)
Yes	0 (0.0%)	7 (38.9%)	12 (9.4%)	0 (0.0%)	0 (0.0%)	0 (0.0%)	1 (3.3%)	20 (6.3%)

Others, procedures with ≤10 specimens including in order abdominal hysterectomy, appendix surgery, craniotomy, gastric surgery, open reduction of fracture, coronary artery bypass graft with only chest incision, hip prosthesis, and breast surgery

*Abbreviations*: *MNGHA* Ministry of National Guard Health Affairs, *KAMC* King Abdulaziz Medical City, *KAH* King Abdulaziz Hospital, *IAFH* Imam Abdulrahman Al Faisal Hospital, *CSEC* Cesarean section, *COLO* Colon surgery, *CBGB* Coronary artery bypass graft with both chest and donor site incisions, *CHOL* Gallbladder surgery, *HER* Herniorrhaphy, *KPRO* Knee prosthesis

^a^Row rather than column percentages were calculated ^b^ Age and gender were calculated for non-duplicate patients

### Causative pathogens

The distribution and rank order of different pathogens by the type of SSI are shown in Table 2. GNPs were the most common (64.2%), followed by GPPs (34.3%) and then fungi (1.4%). The most frequent pathogens were *Staphylococcus aureus* (22.8%), *Pseudomonas aeruginosa* (20.1%), *Klebsiella* spp. (12.2%), *Escherichia coli* (12.2%), *Enterobacter* spp. (7.7%), and *Enterococcus* spp. (5.9%). *Staphylococcus aureus* and *Pseudomonas aeruginosa* were equally the most frequent pathogens in herniorrhaphy and knee prosthesis surgeries. Additionally, *Staphylococcus aureus* was the most frequent pathogen in cesarean section while *Pseudomonas aeruginosa* was the most frequent pathogen in coronary artery bypass graft surgery. *Escherichia coli* was the most frequent pathogen in colon, gallbladder, and other surgeries. In procedures with non-clean wounds, GNPs were more frequent (specially *Pseudomonas aeruginosa)* while GPPs were less frequent (specially *Staphylococcus aureus*, Supplementary data, Table 2B).

**Table 2:** Distribution of pathogens causing surgical site infections (SSIs) in 4 MNGHA hospitals in Saudi Arabia (2007–2016)

	CSEC (*N* = 160)	COLO (*N* = 29)	CBGB (*N* = 194)	CHOL (*N* = 28)	HER (*N* = 21)	KPRO (*N* = 22)	Others (*N* = 38)	Total (*N* = 492)
**All gram positive bacteria**	**89 (55.6%)**	**6 (20.7%)**	**50 (25.8%)**	**6 (21.4%)**	**8 (38.1%)**	**8 (36.4%)**	**2 (5.3%)**	**169 (34.3%)**
*Staphylococcus aureus*	67 (41.9%)	1 (3.4%)	28 (14.4%)	3 (10.7%)	5 (23.8%)	7 (31.8%)	1 (2.6%)	112 (22.8%)
*Enterococcus* spp.	10 (6.3%)	5 (17.2%)	8 (4.1%)	2 (7.1%)	3 (14.3%)	(0.0%)	1 (2.6%)	29 (5.9%)
*Coagulase negative staphylococci*			11 (5.7%)			1 (4.5%)		12 (2.4%)
Other gram positive	12 (7.5%)		3 (1.5%)	1 (3.6%)				16 (3.3%)
**All gram negative bacteria**	**71 (44.4%)**	**23 (79.3%)**	**142 (73.2%)**	**17 (60.7%)**	**13 (61.9%)**	**14 (63.6%)**	**36 (94.7%)**	**316 (64.2%)**
*Acinetobacter* spp*.*	1 (0.6%)	1 (3.4%)	4 (2.1%)	1 (3.6%)		1 (4.5%)	4 (10.5%)	12 (2.4%)
*Pseudomonas aeruginosa*	19 (11.9%)	6 (20.7%)	52 (26.8%)	2 (7.1%)	5 (23.8%)	7 (31.8%)	8 (21.1%)	99 (20.1%)
*Klebsiella spp.*	15 (9.4%)	4 (13.8%)	29 (14.9%)	4 (14.3%)	2 (9.5%)	1 (4.5%)	5 (13.2%)	60 (12.2%)
*Enterobacter* spp*.*	8 (5.0%)	2 (6.9%)	18 (9.3%)	2 (7.1%)	1 (4.8%)	0	7 (18.4%)	38 (7.7%)
*Escherichia coli*	17 (10.6%)	8 (27.6%)	16 (8.2%)	5 (17.9%)	2 (9.5%)	2 (9.1%)	10 (26.3%)	60 (12.2%)
*Serratia* spp*.*	1 (0.6%)		13 (6.7%)	1 (3.6%)				15 (3.0%)
*Proteus* spp*.*	7 (4.4%)		3 (1.5%)		3 (14.3%)			13 (2.6%)
Other gram negative	3 (1.9%)	2 (6.9%)	7 (3.6%)	2 (7.1%)		3 (13.6%)	2 (5.3%)	19 (3.9%)
**Fungi**			**2 (1.0%)**	**5 (17.9%)**				**7 (1.4%)**

*Abbreviations*: As in Table 1. Other gram positive pathogens included *Streptococcus* spp., *Streptococcus beta*-*hemolytic*, and *Streptococcus pneumonia*. Other gram negative pathogens included *Citrobacter* spp., *Bacteroides* spp., *Morganella morganii*, *Burkholderia cepacia*, and *Providencia stuartii*

### Resistant pathogens

Antimicrobial resistance in different pathogens by the type of SSI is shown in Table 3. Approximately 27.7% of GPPs and 16.1% of GNPs were resistant. In GPPs, 30.3% of *Staphylococcus aureus* was methicillin-resistant (MRSA) and 13.0% of *Enterococcus* spp. was vancomycin-resistant (VRE). Approximately 25.0% of the *Klebsiella* spp. were cephalosporin-resistant and 5.5% of *Enterobacteriaceae* were CRE (11.4% in *Klebsiella* spp., 2.0% in *Escherichia coli*, and 0.0% in *Enterobacter* spp.). The highest frequency of MDR in GNPs was seen in *Acinetobacter* spp. (58.3%), followed by *Klebsiella* spp. (20.4%) and *Escherichia coli* (16.3%).

**Table 3:** Antimicrobial resistance in selected pathogens causing surgical site infections (SSIs) in 4 MNGHA hospitals in Saudi Arabia (2007–2016)

	CSEC (*N* = 137)	COLO (*N* = 27)	CBGB (*N* = 155)	CHOL (*N* = 19)	HER (*N* = 18)	KPRO (*N* = 18)	Others (*N* = 36)	Total (*N* = 410)
**Tested pathogens**								
*Staphylococcus* aureus	60 (89.6%)	1 (100.0%)	26 (92.9%)	3 (100.0%)	3 (60.0%)	5 (71.4%)	1 (100.0%)	99 (88.4%)
*Enterococcus* spp.	9 (90.0%)	2 (40.0%)	7 (87.5%)	1 (50.0%)	3 (100.0%)		1 (100.0%)	23 (79.3%)
*Klebsiella* spp. (cephalosporins)	15 (100.0%)	4 (100.0%)	24 (82.8%)	3 (75.0%)	2 (100.0%)	1 (100.0%)	3 (60.0%)	52 (86.7%)
*Enterobacteriaceae*	35 (92.1%)	7 (63.6%)	37 (60.7%)	8 (80.0%)	4 (80.0%)	3 (100.0%)	16 (80.0%)	110 (74.3%)
*Acinetobacter* spp.	1 (100.0%)	1 (100.0%)	4 (100.0%)	1 (100.0%)		1 (100.0%)	4 (100.0%)	12 (100.0%)
*Pseudomonas aeruginosa*	18 (94.7%)	6 (100.0%)	51 (98.1%)	2 (100.0%)	5 (100.0%)	6 (85.7%)	8 (100.0%)	96 (97.0%)
*Klebsiella* spp. (at least 3 classes)	10 (66.7%)	4 (100.0%)	24 (82.8%)	4 (100.0%)	2 (100.0%)	1 (100.0%)	4 (80.0%)	49 (81.7%)
*Escherichia coli*	12 (70.6%)	7 (87.5%)	15 (93.8%)	5 (100.0%)	1 (50.0%)	1 (50.0%)	8 (80.0%)	49 (81.7%)
**Resistance types**								
MRSA	20 (33.3%)	1 (100.0%)	6 (23.1%)	1 (33.3%)	0 (0.0%)	2 (40.0%)	0 (0.0%)	30 (30.3%)
VRE	0 (0.0%)	1 (50.0%)	1 (14.3%)	1 (100.0%)	0 (0.0%)		0 (0.0%)	3 (13.0%)
CephR Klebsiella	1 (6.7%)	1 (25.0%)	4 (16.7%)	2 (66.7%)	2 (100.0%)	1 (100.0%)	2 (66.7%)	13 (25.0%)
CRE	1 (2.9%)	2 (28.6%)	0 (0.0%)	0 (0.0%)	0 (0.0%)	1 (33.3%)	2 (12.5%)	6 (5.5%)
MDR Acinetobacter	0 (0.0%)	1 (100.0%)	2 (50.0%)	1 (100.0%)		1 (100.0%)	2 (50.0%)	7 (58.3%)
MDR Pseudomonas	0 (0.0%)	1 (16.7%)	4 (7.8%)	0 (0.0%)	0 (0.0%)	1 (16.7%)	1 (12.5%)	7 (7.3%)
MDR Klebsiella	0 (0.0%)	1 (25.0%)	3 (12.5%)	1 (25.0%)	2 (100.0%)	1 (100.0%)	2 (50.0%)	10 (20.4%)
MDR Escherichia coli	2 (16.7%)	2 (28.6%)	0 (0.0%)	2 (40.0%)	0 (0.0%)	0 (0.0%)	2 (25.0%)	8 (16.3%)

*Abbreviations*: As in Table 1. Tested pathogens referred to pathogens tested out of pathogens causing SSI; resistance was presented out of pathogens tested; *MRSA* Methicillin-resistant *Staphylococcus* aureus, *VRE* Vancomycin-resistant *Enterococcus*, *CephR Klebsiella* Cephalosporin resistant *Klebsiella*, *CRE* Carbapenem resistant *Enterobacteriaceae*, *MDR* Multidrug resistant gram negative pathogens that tested non-susceptible (resistant or intermediate) to at least one agent in at least 3 out of 5 antimicrobial classes (see Methods)

**Fig. 1:**
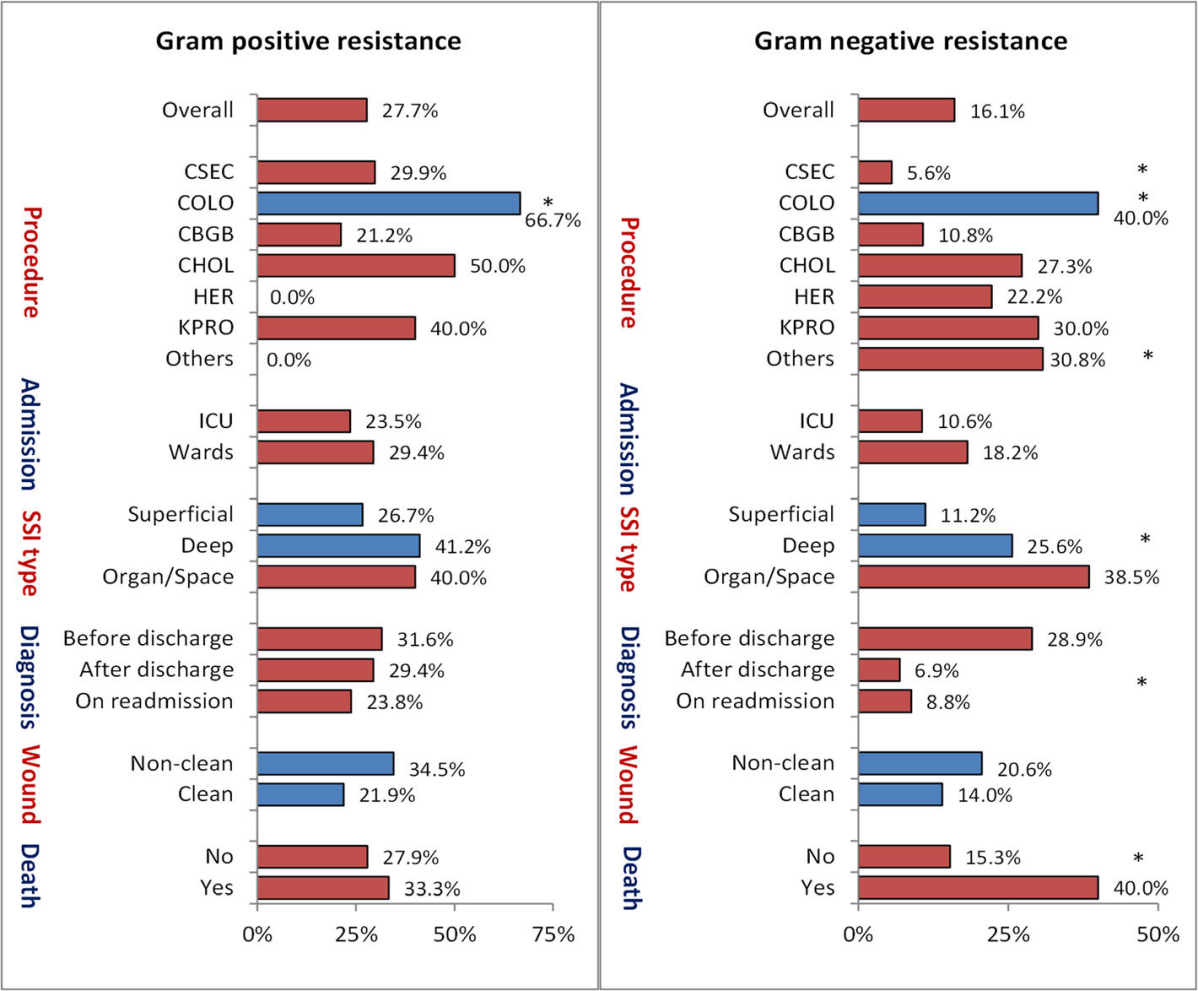
Overall resistance rates of pathogens causing surgical site infections (SSIs) by clinical characteristics in 4 MNGHA hospitals in Saudi Arabia (2007–2016). Note: Gram positive resistance includes MRSA or VRE. Gram-negative resistance include CephR Klebsiella, CRE, MDR Acinetobacter, MDR Pseudomonas, MDR Klebsiella, or MDR E-coli, as shown in Table 3. * indicate significant differences

The distributions of overall resistance by clinical characteristics are shown in Fig. 1. Resistant GPPs (including MRSA or VRE) showed some variability by the type surgery done, being highest with colon surgery (*p* = 0.013). However, there was no significant variability in resistant GPPs by the type of admission, wound class, diagnostic types of SSI, time of diagnosis, nor hospital mortality. Resistant GNPs (including cephalosporin-resistant *Klebsiella* spp., CRE, MDR *Acinetobacter* spp., MDR *Pseudomonas aeruginosa*, MDR *Klebsiella* spp., or MDR *Escherichia coli*) showed significant variability with all examined characteristics with the exception of the wound class and type of admission. For example, resistant GNPs were significantly higher with colon surgery but lower with cesarean section compared with all other surgeries combined. Additionally, resistant GNPs were significantly associated with higher mortality, pre-discharge diagnosis of SSI, and with increasing the depth of SSI. None of the different types of resistance was different by the procedure wound class (Supplementary data, Table 3B).

### Comparisons with NHSN

**Fig. 2:**
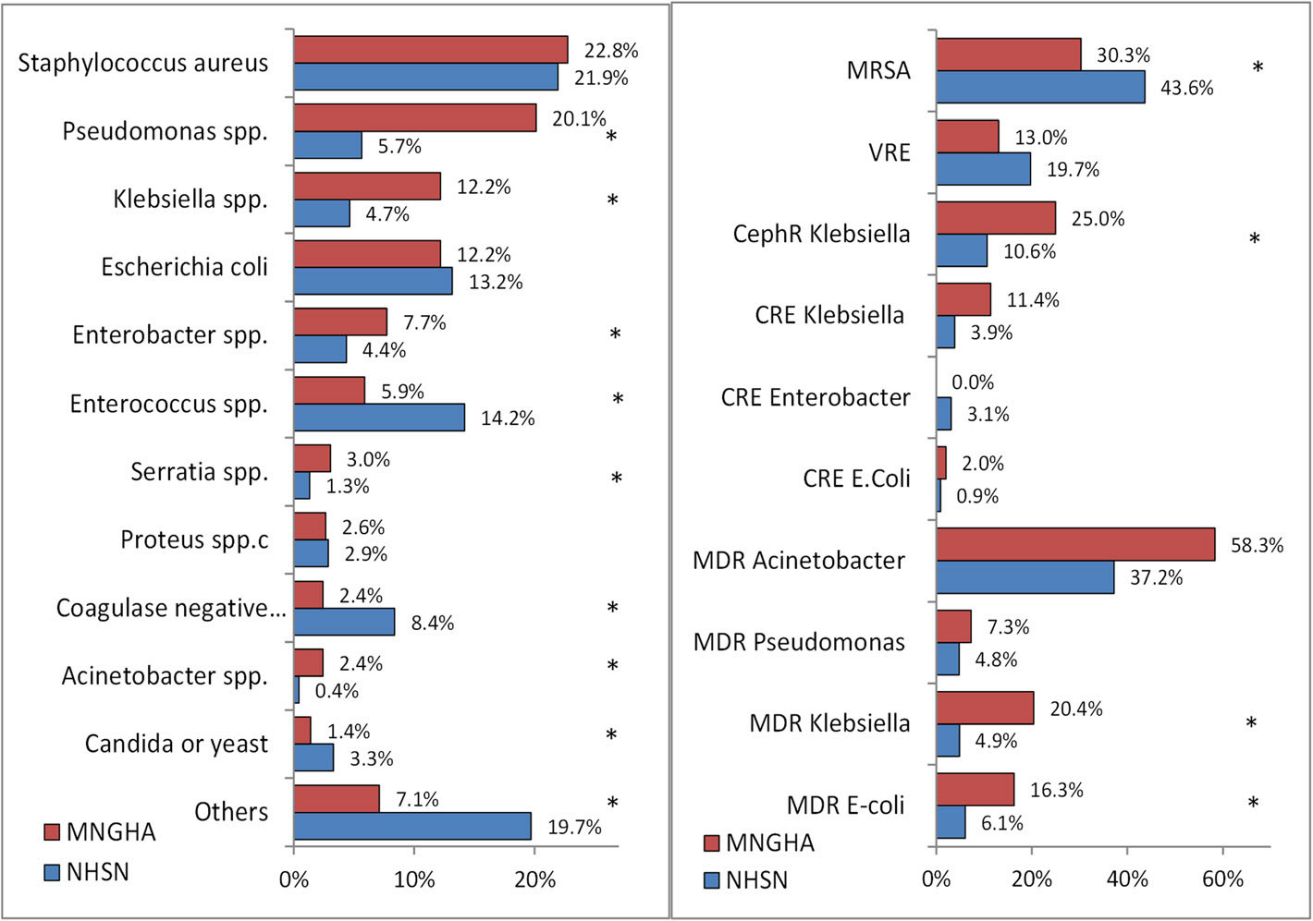
Comparisons of the percentage of pathogens causing SSI (left) and their resistance of patterns (right*) in MNGHA hospitals (2007–2016) and NHSN hospitals (2009–2014). Abbreviations: As in Table 3; SSI, surgical site infections. NHSN rates were based on the two published NHSN reports (references [13, 14]). NHSN VRE rate shown was the rate of both *Enterococcus faecalis* and *Enterococcus faecium* combined. * indicate significant differences

The distribution of pathogens and their resistance patterns in MNGHA compared with NHSN hospitals are shown in Fig. 2. *Pseudomonas aeruginosa*, *Klebsiella* spp., *Enterobacter* spp., *Serratia* spp., and *Acinetobacter* spp. were significantly more frequent while *Enterococcus*, *Coagulase negative staphylococci*, and fungi were significantly less frequent in MNGHA hospitals compared with NHSN hospitals. MRSA was significantly less frequent while cephalosporin-resistant Klebsiella spp., MDR Klebsiella spp., and MDR *Escherichia coli* were significantly more frequent in MNGHA hospitals compared with NHSN hospitals.

## Discussion

The current study shows the distribution of SSI pathogens and their resistance patterns in 6 adult surgical procedures performed over 10 years in a multi-hospital healthcare system located in a high income Middle Eastern country. A number of points should be highlighted while we are interpreting the current findings; they represent only laboratory-confirmed SSI events, as 33% of all SSI events were diagnosed clinically without laboratory confirmation. The data were collected in the presence of a local guideline for surgical prophylaxis consistent with international standards [19]. Additionally, surgical bundle of the Institute for Healthcare Improvement (IHI) was implemented throughout the study [20]. Compliance with the guideline and the surgical bundle, however, has varied widely between surgeries and from time to time (data not shown).

While it is challenging to compare the distribution of SSI pathogens between different studies covering different surgical procedures, *Staphylococcus aureus* was the most frequent pathogen in the current study as well as studies done in developed [5, 8, 9] and developing countries [1, 21]. For example, it was approximately 23% in the current study compared with 20 to 30% in these studies [1, 5, 8, 9]. However, GNPs were more prevalent in the current study (64%) than seen in developed countries (36–46%) [5, 8, 9]. The prevalence of *Pseudomonas aeruginosa* and less extent *Klebsiella* spp*.* were several folds higher while *Escherichia coli* was generally similar to those of the US and European hospitals, even after considering surgery-specific data [5, 8, 9]. The high contribution of GNPs in the current study was consistent with several reports from Saudi Arabia [10–12] and developing countries [1], that showed contribution rates between 55 and 77%. This may be explained by inadequate hand hygiene practices [22] and high environmental burden of GNPs, that are usually more resistant to disinfectants compared with GPPs [23]. However, the high contribution of GNPs may not be simply explained by the difference in the proportions of included procedures as the surgery-specific prevalence of *Pseudomonas aeruginosa* and *Klebsiella* spp. in the current study was much higher than corresponding NHSN figures for abdominal, pelvic, cardiac, and orthopedic procedures [8, 9]. Additionally, it is unlikely to reflect differential antimicrobial selection pressure as the local guidelines for antimicrobial prophylaxis were consistent with international standards [19].

In addition to the low contribution, GPPs causing SSI in the current study were generally less resistant than reported internationally. For example, MRSA rates was 30% in the current study compared with more than 40% in USA [8, 9], Europe [5], and Asia [24, 25]. In Saudi Arabia, there has been a great variability in the published MRSA rates, probably due to mixing community and hospital isolates as well as clinical and surveillance isolates [26, 27]. Nevertheless, the current finding was consistent with the local MRSA rates obtained from HAI specimens (mainly wound), which ranged between 16 and 57% [26]. For VRE, the current rates (13%) was comparable to recent reports from Saudi Arabia [28]. Additionally, it was comparable to international rates [5, 24], with exception of North and South American ones which traditionally have very high rates of VRE [8, 9, 24]. Despite the relatively low resistance of GPPs observed in the current and local studies, reports has warned from an increasing trend in the resistance of GPPs in Saudi Arabia, specially VRE, probably due to overuse of broad-spectrum antimicrobials and development of new resistance patterns [27, 28].

In addition to higher contribution, GNPs causing SSI in the current study were generally more resistant than reported in US hospitals. For example, all GNPs in the current study (with exception of *Enterobacter* spp.) were more resistant than NHSN hospitals, with significant difference in cephalosporin-resistant *Klebsiella* spp., MDR *Klebsiella* spp., and MDR *Escherichia coli* [8, 9]. On the other hand, the current rates were even lower than the extremely high rates of cephalosporin and carbapenem resistance among GNPs reported in some developing countries such as Egypt [13], India [29], and Iran [14]. The high rate of resistance in GNPs in the current study is probably reflecting a wide range of resistance mechanisms in GNPs observed in our hospitals, such as NDM, OXA 48 and MGrb and outer membrabe protein (OMP) resistance [30–34]. The high rate of resistance in GNPs in the current study is quite alarming as it already increased the mortality by 25%. Additionally, this can be used as a justification for initiation and continuation of broad-spectrum antibiotics, leading to a vicious cycle of enhancing resistance. Consistent with current data, a local study showed that 77% of pathogens isolated from SSI after abdominal surgery were resistant to the prophylactic antibiotic given preoperatively [11].

With the limited data available locally and regionally, this report can serve as a unique benchmark for caregivers engaged in SSI prophylaxis and antimicrobial stewardship programs. The data were prospectively collected over 10 years by well-trained infection preventionists in 4 hospitals, using the same standard surveillance methodology and the same SSI preventive practices. The relatively large sample size allowed for presenting surgery-specific pathogen distribution and resistance patterns. The use of NHSN resistance definitions allowed for previously unmatched comparisons of resistance patterns between the two differently-matured healthcare systems. Nevertheless, few limitations are acknowledged. The analyzed data were only a sample of a much bigger number of surgeries done in the 4 hospitals during the period covered by the study and almost one-third of SSI were diagnosed clinically. However, these are typical for all studies following the NHSN definitions and NHSN-recommended targeted surveillance methodology. As in other similar studies, underestimation of SSI diagnosis cannot be excluded. However, this should be unlikely, as our population is entitled to free care in our hospitals, which make the likelihood of our patients seeking medical care elsewhere is very low. Despite being beyond of the scope of this paper, the lack of extensive data analysis on the colonization rates and the risk factors that can possibly affect the resistance patterns limit the interpretation of the current findings.

## Conclusions

*Staphylococcus aureus* remains the most frequent SSI pathogen, with 30% are MRSA. GNPs are responsible for approximately 64% of SSI and were generally more resistant than seen in Western countries. Resistant GNPs were associated with increased mortality. Making this information available to caregivers and healthcare leaders is critical to secure resources and ensure support in implementing interventions, such as antimicrobial stewardship programs and evidence-based SSI preventive practices [6].

## Supplementary information

**Additional file 1: Table-2B.** Distribution of pathogens causing surgical site infections (SSIs) by wound class in 4 MNGHA hospitals in Saudi Arabia (2007–2016). **Table-3B.** Antimicrobial resistance in selected pathogens causing surgical site infections (SSIs) by wound class in 4 MNGHA hospitals in Saudi Arabia (2007–2016).

## Data Availability

The datasets used and/or analyzed during the current study are available from the corresponding author on reasonable request.

## Abbreviations

CBGB: Coronary artery bypass graft with both chest and donor site incisions
CephR: Cephalosporin resistant
CHOL: Gallbladder surgery
COLO: Colon surgery
CRE: Carbapenem-resistant Enterobacteriaceae
CSEC: Cesarean section
GCC: Gulf Cooperation Council
GNPs: Gram negative pathogens
GPPs: Gram positive pathogens
HAIs: Healthcare-associated infections
HER: Herniorrhaphy
IABFH: Imam Abdulrahman Bin Faisal Hospital
IHI: Institute for Healthcare Improvement
KAH: King Abdulaziz Hospital-Alhassa
KAMC-J: King Abdulaziz Medical City-Jeddah
KAMC-R: King Abdulaziz Medical City-Riyadh
KPRO: Knee prosthesis
MDR: Multidrug resistance
MNGHA: Ministry of National Guard Health Affairs
MRSA: Methicillin-resistant *staphylococcus aureus*
NHSN: US National Healthcare Safety Network
SSI: Surgical site infection
VRE: Vancomycin-resistant enterococcus
