# Supplementary material for: High contribution and impact of resistant gram negative pathogens causing surgical site infections at a multi-hospital healthcare system in Saudi Arabia, 2007–2016
Source: BMC Infect Dis. 2020 Apr 7;20:275. doi: 10.1186/s12879-020-4939-6 (PMC7140359; doi:10.1186/s12879-020-4939-6)
Supplement: Supplementary file 1 — Additional file 1: Table-2B. Distribution of pathogens causing surgical site infections (SSIs) by wound class in 4 MNGHA hospitals in Saudi Arabia (2007–2016). Table-3B. Antimicrobial resistance in selected pathogens causing surgical site infections (SSIs) by wound class in 4 MNGHA hospitals in Saudi Arabia (2007–2016). [file 12879_2020_4939_MOESM1_ESM.docx]

**Supplementary data**

**Table-2B: Distribution of pathogens causing surgical site infections (SSIs) by wound class in 4 MNGHA hospitals in Saudi Arabia (2007-2016)**

|  | **Clean**  (N=312) | **Non-clean**  (N=180) | **Total**  (N=492) | **p-value** |
| --- | --- | --- | --- | --- |
| **All gram positive bacteria** | **93 (29.8%)** | **76 (42.2%)** | **169 (34.3%)** | 0.006 |
| *Staphylococcus aureus* | 62 (19.9%) | 50 (27.8%) | 112 (22.8%) | 0.046 |
| *Enterococcus* spp. | 14 (4.5%) | 15 (8.3%) | 29 (5.9%) | 0.086 |
| *Coagulase negative staphylococci* | 12 (3.8%) | 0 (0.0%) | 12 (2.4%) | 0.005 |
| Other gram positive* | 5 (1.6%) | 11 (6.1%) | 16 (3.3%) | 0.007 |
| **All gram negative bacteria** | **211 (67.6%)** | **92 (51.1%)** | **303 (61.6%)** | <0.001 |
| *Acinetobacter spp.* | 9 (2.9%) | 3 (1.7%) | 12 (2.4%) | 0.547 |
| *Pseudomonas aeruginosa* | 78 (25.0%) | 21 (11.7%) | 99 (20.1%) | <0.001 |
| *Klebsiella spp.* | 40 (12.8%) | 20 (11.1%) | 60 (12.2%) | 0.534 |
| *Enterobacter spp.* | 26 (8.3%) | 12 (6.7%) | 38 (7.7%) | 0.474 |
| *Escherichia coli* | 33 (10.6%) | 27 (15.0%) | 60 (12.2%) | 0.159 |
| *Serratia spp.* | 13 (4.2%) | 2 (1.1%) | 15 (3.0%) | 0.054 |
| *Proteus spp.* | 6 (1.9%) | 7 (3.9%) | 13 (2.6%) | 0.245 |
| Other gram negative* | 12 (3.8%) | 7 (3.9%) | 19 (3.9%) | >0.99 |
| **Fungi** | **2 (0.6%)** | **5 (2.8%)** | **7 (1.4%)** | **0.106** |

Abbreviations: Pathogens of non-clean wounds included 174 from clean-contaminated wounds, 1 from contaminated wound, and 5 from dirty wounds. Other gram positive pathogens included *Streptococcus* spp. , *Streptococcus* *beta*-*hemolytic*, and *Streptococcus* *pneumonia*. Other gram negative pathogens included *Citrobacter* spp., *Bacteroides* spp., *Morganella* *morganii*, *Burkholderia* *cepacia*, and *Providencia* *stuartii.*

**Supplementary data**

**Table-3B: Antimicrobial resistance in selected pathogens causing surgical site infections (SSIs) by wound class in 4 MNGHA hospitals in Saudi Arabia (2007-2016)**

|  | **Clean**  (N=312) | **Non-clean**  (N=180) | **Total**  (N=492) |  |
| --- | --- | --- | --- | --- |
| **Tested pathogens** |  |  |  |  |
| *Staphylococcus* aureus | 53 (85.5%) | 46 (92.0%) | 99 (88.4%) | 0.285 |
| *Enterococcus* spp. | 13 (92.9%) | 10 (66.7%) | 23 (79.3%) | 0.169 |
| *Klebsiella* spp. (cephalosporins) | 33 (82.5%) | 19 (95.0%) | 52 (86.7%) | 0.249 |
| *Enterobacteriaceae* | 66 (69.5%) | 44 (83.0%) | 110 (74.3%) | 0.071 |
| *Acinetobacter* spp. | 9 (100.0%) | 3 (100.0%) | 12 (100.0%) | --- |
| *Pseudomonas* *aeruginosa* | 75 (96.2%) | 21 (100.0%) | 96 (97.0%) | >0.99 |
| *Klebsiella* spp. (at least 3 classes) | 30 (75.0%) | 19 (95.0%) | 49 (81.7%) | 0.081 |
| *Escherichia* *coli* | 27 (81.8%) | 22 (81.5%) | 49 (81.7%) | >0.99 |
| **Resistance types** |  |  |  |  |
| MRSA | 13 (24.5%) | 17 (37.0%) | 30 (30.3%) | 0.180 |
| VRE | 1 (7.7%) | 2 (20.0%) | 3 (13.0%) | 0.560 |
| CephR Klebsiella | 7 (21.2%) | 6 (31.6%) | 13 (25.0%) | 0.510 |
| CRE | 4 (6.1%) | 2 (4.5%) | 6 (5.5%) | >0.99 |
| MDR Acinetobacter | 5 (55.6%) | 2 (66.7%) | 7 (58.3%) | >0.99 |
| MDR Pseudomonas | 6 (8.0%) | 1 (4.8%) | 7 (7.3%) | >0.99 |
| MDR Klebsiella | 7 (23.3%) | 3 (15.8%) | 10 (20.4%) | 0.720 |
| MDR Escherichia coli | 2 (7.4%) | 6 (27.3%) | 8 (16.3%) | 0.117 |

Abbreviations: Pathogens of non-clean wounds included 174 from clean-contaminated wounds, 1 from contaminated wound, and 5 from dirty wounds. Tested pathogens referred to pathogens tested out of pathogens causing SSI; resistance was presented out of pathogens tested; MRSA, methicillin-resistant *Staphylococcus* aureus; VRE, vancomycin-resistant *Enterococcus*; CephR *Klebsiella*, cephalosporin resistant *Klebsiella*; CRE, carbapenem resistant *Enterobacteriaceae*; MDR, multidrug resistant gram negative pathogens that tested non-susceptible (resistant or intermediate) to at least one agent in at least 3 out of 5 antimicrobial classes (see methods).
